# The Influence of SPI 7S and 11S on the Stability of *Lonicera caerulea* L. Anthocyanins and Interaction Mechanism with C3G

**DOI:** 10.3390/foods15111933

**Published:** 2026-05-29

**Authors:** Yingying Zhou, Yixin Yuan, Zhicong Wang, Di Wu, Yinan Du, Jiangning Hu

**Affiliations:** 1 State Key Laboratory of Marine Food Processing and Safety Control, Dalian Polytechnic University, Dalian 116034, China; 15238225546@163.com (Y.Z.);; 2 National Engineering Research Center of Seafood, School of Food Science and Technology, Dalian Polytechnic University, Dalian 116034, China

**Keywords:** soy protein isolate, anthocyanins, interaction, molecular mechanism

## Abstract

This study examined the effects of soy protein isolate (SPI) 7S and SPI 11S on the anthocyanin (AN) retention rate of *Lonicera caerulea* L. under different processing conditions and further analyzed the molecular interaction mechanisms between 7S/11S and cyanidin-3-O-glucoside (C3G). The results demonstrated that SPI increased the retention rate of anthocyanins to varying degrees while also enhancing the digestive stability. Multispectral results indicated that static quenching occurred between 7S/11S and C3G, and the polarity changes in the amino acid microenvironment varied with pH. Thermodynamic analysis indicated that hydrogen bonds dominated the interaction under both pH conditions, while a certain degree of hydrophobic interaction was additionally observed under neutral conditions. After binding with C3G, the proportion of β-sheet structures in SPI decreased and the proportion of other structures increased. Finally, molecular docking further simulated the binding between SPI and C3G and revealed the important roles of hydrophobic interactions and hydrogen bonding, which also promoted the combination of SPI and C3G to form a stable complex. This study provides a mechanistic reference for using proteins as effective carriers to protect anthocyanins, with implications for developing functional food components with enhanced stability.

## 1. Introduction

*Lonicera caerulea* L. is a plant of the Caprifoliaceae family native to Northeast Asia [1]. *Lonicera caerulea* L. is highly nutritious, with a higher anthocyanin content than commonly studied fruits such as blueberry, particularly the most representative compound, cyanidin-3-O-glucoside (C3G) [2]. Anthocyanins (ANs) are widely found in fruits and vegetables; they are water-soluble flavonoid compounds that can serve as natural pigments [3]. They possess a variety of bioactive properties, including anticancer, anti-inflammatory and antioxidant effects [4]. Therefore, ANs are often added to food and cosmetics as natural colorants or functional components [5]. However, ANs is unstable, and they are highly susceptible to degradation under various processing and storage conditions, such as heating, exposure to light and changes in pH [6]. In particular, among these factors, changes in pH have the most significant effect on the stability of ANs. Under acidic condition, the primary form of ANs is the stable flavylium cation, which shows bright color and is commonly found in acidic systems such as fruit juices. However, in a neutral environment, ANs are converted into colorless chalcone and other derivatives. This transformation leads to discoloration and a sharp decrease in stability, thereby limiting their application in functional foods [7]. Therefore, it is worthwhile to explore how to improve the retention rate of anthocyanins under varying processing conditions.

Recently, there has been a growing focus on the strategy of forming complexes between the food matrix and ANs through noncovalent interactions to enhance their stability. This approach is simple, uses natural materials and preserves the biological activity of ANs. Noncovalent interactions such as van der Waals forces, hydrophobic interactions and hydrogen bonds can cause certain components in food, such as polysaccharides and proteins, to bind to ANs [8,9,10]. These interactions increase the stability of ANs against thermal treatment, light exposure and gastrointestinal digestion. Among these components, proteins are considered one of the most promising stabilizing carriers because of their functional properties and flexible molecular structure. Previous studies have shown that bovine serum albumin can form complexes with blueberry anthocyanins, thereby enhancing the stability of the anthocyanins [11]. Zang et al. [12] reported that the antioxidant activity and stability of blueberry anthocyanins were enhanced by whey protein isolate (WPI). Ma et al. [13] further demonstrated that proteins can form a protective microenvironment during in vitro digestion, thereby improving the bioavailability of ANs. Therefore, the mechanisms of interaction between anthocyanins and food proteins are essential for developing effective stabilization strategies.

Among various food proteins, soybean protein isolate (SPI) has attracted considerable attention. SPI is widely available and cost-effective. It also exhibits desirable functional properties, such as emulsifying ability, gelling capacity and film-forming properties [14,15,16]. SPI molecules contain abundant hydroxyl groups, amino groups and aromatic residues. Therefore, SPI can interact with polyphenols through multiple interactions, which improves the stability of Ans. Reference [3] showed that SPI can improve the retention rate of blueberry ANs during processing and storage. The main components of SPI are 7S (β-conglycinin) and 11S (glycinin), which account for approximately 70% [17]. Both 7S and 11S exhibit high structural stability and good processing adaptability. These two proteins contain abundant polar groups and hydrophobic regions. Although the general interaction between SPI and anthocyanins has been widely reported, few studies have examined the effects of its two major globulins, 7S and 11S, on anthocyanins under different processing conditions. And the protective effects of 7S and 11S on *Lonicera caerulea* L. anthocyanins and the underlying mechanisms at the molecular level have not been reported.

Therefore, this study seeks to clarify the distinct protective roles of the 7S and 11S fractions from SPI in stabilizing *Lonicera caerulea* L. anthocyanins at pH 3.0 and pH 7.0. The stability of 7S/11S–ANs complexes against heat, light and the gastrointestinal environment was evaluated. Various spectroscopic techniques, such as fluorescence and infrared spectroscopy, were employed to investigate the mechanisms of action of C3G and 7S/11S, and structural changes in the proteins were also analyzed. Molecular docking was further performed to show the detailed binding methods of the complex system. This study offers a theoretical groundwork for designing safe, natural and processing-adaptable ANs stabilization platforms, based on elucidation of how plant proteins interact with ANs at the molecular level.

## 2. Materials and Methods

### 2.1. Materials

*Lonicera caerulea* L. extract (ANs content 25%) was purchased from Chengdu Pusi Biotechnology Co., Ltd. (Chengdu, China), C3G (purity 98%) was acquired from Nanjing Yuanzhi Biotechnology Co., Ltd. (Nanjing, China), SPI 7S and 11S were homemade in the laboratory. Reagents were of analytical purity.

### 2.2. Protein Extraction and Validation

SPI 7S and 11S were prepared using the extraction method described by Wang et al. [18]. The low-temperature defatted soybean meal was mixed with ethanol at a ratio of 5:1 (*v*/*w*) for 2 h, followed by drying at 25 °C. The ethanol-washed low-temperature defatted soybean meal was ground into a powder, mixed with distilled water at a ratio of 1:10 (*w*/*v*), and stirred for 2 h. The insoluble residue was removed by filtration, and the filtrate was centrifuged at 9000 *g* for 30 min. The supernatant was adjusted to a sodium bisulfite concentration of 0.989 g/L, thoroughly mixed, and allowed to stand for 30 min. The pH of the solution was adjusted to 6.4, and the mixture was left to stand overnight at 4 °C. After standing, the solution was centrifuged at 6500 *g* for 20 min, and the resulting precipitate was redissolved. The pH was adjusted to 7.0, and the redissolved solution was freeze-dried to obtain glycinin (11S). To the supernatant obtained after standing and centrifugation, NaCl was added to adjust the ionic concentration to 0.25 M. After stirring to dissolve, the pH of the solution was adjusted to 4.9. The resulting solution was left to stand at 4 °C for 1 h and then centrifuged at 6500 *g* for 20 min. The supernatant was diluted with an equal volume of pre-chilled distilled water (4 °C), and the pH was adjusted to 4.8. After centrifugation at 6500 *g* for 20 min, the precipitate was redissolved, the pH was adjusted to 7.0, and freeze-drying was performed to obtain β-conglycinin (7S).

The BCA assay kit was employed to determine the purity of the proteins, with the purity of 7S and 11S being 92.98 ± 0.58% and 97.13 ± 0.86%, respectively. The extracted proteins were verified using the SDS-PAGE method described by Din et al. [19]. The protein extract was diluted with distilled water to 4 mg/mL, and the diluted extract was mixed with an equal volume of SDS sample buffer, then heated at 96 °C for 5–8 min. The solution was cooled to room temperature. A 10% resolving gel and a 3.0% stacking gel were used. Ten microliters of each sample were loaded onto the SDS-PAGE gel. The samples were electrophoresed at a constant voltage of 80 V for 20 min, then the voltage was adjusted to 120 V for approximately 1 h. After electrophoresis, the gel was stained with Coomassie Brilliant Blue R250 (0.05%, *w*/*v*) in a methanol-acetic acid–water mixture (25:10:65 *v*/*v*/*v*), and then destained in the same solution without the dye. The solution was replaced two to three times with distilled water for destaining. Subsequently, the prepared gel was scanned using a chemiluminescence imaging analyzer (ChemiDoc Touch, Bio-Rad Laboratories, Inc., Hercules, CA, USA). The result is shown in Figure S1. By comparing the obtained banding patterns with those reported in relevant stugies [19], it was confirmed that the extracted proteins were 7S and 11S.

### 2.3. Stability Testing

ANs were weighed and dissolved in citrate-sodium citrate buffer (pH 3.0) and phosphate-buffered saline (PBS, pH 7.0), resulting in a final concentration of 2 mg/mL. SPI 7S and 11S fractions were also dissolved in the above buffers to prepare protein solutions at different concentrations (0.2, 0.4, 1, 2 mg/mL). ANs solutions and protein solutions were mixed at equal volumes. Ultimately, the mass ratios of ANs to proteins in the solution were 1:1, 2:1, 5:1, 10:1 and 1:0, respectively.

Thermal stability was determined by subjecting the samples to thermal treatment at 85 °C in the dark for 4 h. Samples were collected every hour to determine changes in anthocyanin content. For the photostability assay, the freshly prepared samples were exposed to UV light (365 nm) for 4 d. Samples were collected every day to determine changes in anthocyanin content.

The collected samples were mixed with two different buffers (pH 1 and pH 4.5). The absorbance of the samples was then measured at wavelengths of 520 nm and 700 nm after reacting for 1 h at room temperature in the dark [20]. ANs content was calculated from the absorbance values by means of the following equation:

(1) A = (A_520nm_ − A_700nm_) pH1.0 − (A_520nm_ − A_700nm_) pH4.5,

(2) C(mg/mL) = (A × DF × MW)/(ε × L),

where A stands for the absorbance, DF is the dilution factor, the values of MW and ε are 449.2 g/mol and 26,900 L·mol^−1^·cm^−1^, respectively, and they represent the molecular weight and molar extinction coefficient of C3G, respectively, and the value of L is 1 cm, representing the path length.

The retention rate of ANs was calculated according to Equation (3).

(3) Retention Rate (%) = (Treated anthocyanins content/Original anthocyanins content) × 100.

### 2.4. In Vitro Gastrointestinal Digestion

Slight modifications were made to the in vitro simulated gastrointestinal digestion experiment, which was reported by Yan et al. [21]. ANs and SPI 7S/11S were dissolved in PBS (pH 7) and fully hydrated, respectively. An equal volume of ACN solution and SPI solution was then mixed and shaken well. The concentration ratio was determined according to the previous stability results. Based on an analysis of the previous results regarding machining stability, the mass ratios of ANs to proteins in the solution were 2:1. In addition, an equal concentration of C3G solution without protein was prepared as the control.

2 M HCl solution was employed to alter the pH of the samples to 2.0, then pepsin was added, and pepsin accounts for 4% of the total protein mass. The enzyme content of the control group was consistent with that of the protein group. At 37 °C, the solution was stirred continuously for 2 h to simulate gastric digestion. Subsequently, 1 M NaOH was employed to alter the pH of the samples to 7.0. The mass of trypsin was also 4% of the protein mass in the solution, and the control group was the same. The mixture was digested at 37 °C for 4 h with continuous stirring. Samples were collected hourly during the digestion process and boiled for 5 min to inactivate the enzymes.

### 2.5. Fluorescence Spectroscopy

A fluorescence spectrophotometer (F-4700, Hitachi Ltd., Tokyo, Japan) was utilized for measuring the intrinsic fluorescence spectra of the samples. PBS and citrate buffer (pH 3.0) were employed to prepare C3G and SPI solutions. The 7S/11S solution was mixed with equal volumes of C3G solutions at different concentrations. C3G concentrations ranged from 0 to 50 μmol/L (0, 10, 20, 30, 40 and 50 μmol/L), with a fixed protein concentration of 10 μmol/L in the mixture. The measurements were executed at 298, 303 and 308 K. The experimental parameters were adjusted based on previous experiments [13]. The scanning was conducted under the conditions of a voltage of 400 V, an excitation wavelength of 280 nm, and an emission wavelength ranging from 300 to 500 nm. The slit width was 10 nm, and the scanning rate was 3000 nm/min.

### 2.6. Fluorescence Quenching Mechanism

Firstly, to evaluate the potential influence of the inner filter effect (IFE) caused by the light absorption of C3G, UV–visible absorption spectra of C3G were recorded at the same concentrations used for fluorescence measurements. As shown in Figure S2, the absorbance of C3G at 10–50 μM was sufficiently low, indicating that the IFE did not significantly interfere with the fluorescence quenching analysis.

The fluorescence quenching rate constant of the system was determined using the Stern–Volmer equation below, which in turn allowed the quenching mechanism to be identified [22].

(4) F_0_/F = 1 + K_SV_Q = 1 + K_q_τ_0_Q,

In the equation, F_0_ and F denote the protein fluorescence intensities before and after the addition of C3G, respectively. Kq is the quenching rate constant, while K_SV_ represents the Stern–Volmer quenching constant. Q refers to the C3G concentration, and τ_0_ is the fluorescence lifetime of the protein, which was taken as 10^−8^ s.

The double-logarithmic Stern–Volmer equation was employed to determine the binding constant and the number of binding sites of the complex [23]. The equation is as follows:

(5) Log [(F_0_ − F)/F] = logK_a_ + nlogQ,

where Ka represents the binding constant, and *n* represents the number of binding sites.

Calculate thermodynamic parameters using Equations (6) and (7) [24]:

(6) lnK_a_ = − (ΔH/RT) (ΔS/R),

(7) ΔG = ΔH − TΔS,

In this equation, ΔG, ΔS and ΔH represent the changes in free energy, entropy and enthalpy associated with the binding process, respectively. Ka is the binding constant obtained from Equation (5), T is the absolute temperature, and R is the gas constant (8.314 J·mol^−1^·K^−1^).

### 2.7. Circular Dichroism (CD) Spectroscopy

SPI 7S/11S and C3G were separately dissolved in citrate–citric acid buffer and PBS. The SPI solution was then mixed with equal volumes of C3G solutions at different concentrations and vortexed thoroughly. SPI–C3G complex solutions were prepared at mass ratios of 1:0, 1:1, 1:2 and 1:5 (SPI: C3G). CD spectra of the diluted samples were obtained using a circular dichroism spectrometer (J-1500, JASCO, Hachioji, Tokyo, Japan). The measurement temperature was 298 K. The wavelength range and scanning speed for the sample were 190–260 nm and 100 nm/min, respectively. With the help of the instrument software, the CD spectra were analyzed to determine the secondary structure composition of the protein [25].

### 2.8. Fourier-Transform Infrared (FT-IR) Spectroscopy

SPI and C3G solutions at different concentrations were prepared in citrate–citric acid buffer and PBS at room temperature. The solutions were mixed at equal volumes. The samples were freeze-dried after pre-freezing at −80 °C. The obtained powder samples were mixed with spectroscopic-grade potassium bromide in a 1:100 ratio and compressed into a thin pellet. FT-IR spectra were recorded using a Fourier-transform infrared spectrometer (Frontier, PerkinElmer Inc., Waltham, MA, USA) operating at a resolution of 4 cm^−1^, with 64 scans, over a scanning range of 400–4000 cm^−1^ [24].

### 2.9. Molecular Docking

The UniProt database (https://www.uniprot.org/uniprotkb, accessed on 1 February 2026) and the RCSB PDB database (https://www.rcsb.org/, accessed on 1 February 2026) were used to obtain the information regarding 7S (3AUP) and 11S (1OD5) [26]. Discovery Studio 2019 was used to import the obtained structures for protein structure optimization. In Discovery Studio 2019, the “Prepare Protein” module was used. Through pKa prediction, the protonation states of amino acid residues (such as His, Asp, and Glu) were adjusted at pH 3.0 and 7.0. This process included removing water molecules, assigning charges, adding hydrogen atoms and completing missing amino acids and side chains. The optimized protein structures were then saved as PDB file format. The structural information of the small molecules was obtained from the PubChem database. These structures were energy-minimized in Discovery Studio 2019. At pH 3.0, C3G was docked as the positively charged flavylium cation. At pH 7.0, it was adjusted to the neutral form according to the equilibrium state. Then, they were exported in PDB format. Protein and ligand PDBQT files were prepared using AutoDock 4.0, and AutoDock Vina 1.2.6 was employed for molecular docking [27]. The results were finally screened based on the highest clustering frequency and binding affinity. The results were visualized and analyzed using PyMOL 3.1 and Discovery Studio 2019 [28].

### 2.10. Statistical Analysis

Each experimental procedure was repeated three times, and the results are expressed as the mean ± standard deviation (SD). A one-way analysis of variance (ANOVA) was performed, and statistical significance was defined as *p* < 0.05.

## 3. Results and Discussion

### 3.1. Stability of ANs Influenced by 7S or 11S

**Figure 1:**
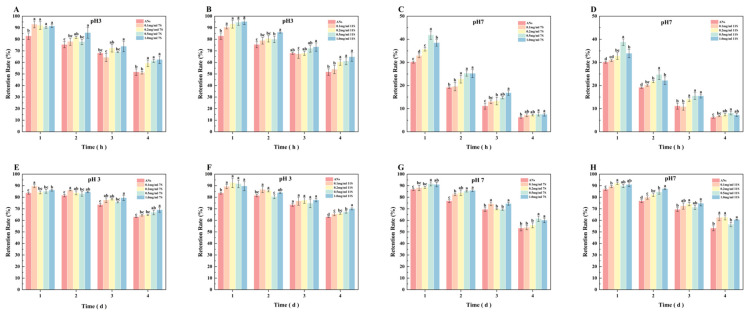
Retention rates of *Lonicera caerulea* L. anthocyanins with and without SPI 7S and 11S after heating (pH 3 (**A**,**B**), pH 7 (**C**,**D**)) and light exposure (pH 3 (**E**,**F**), pH 7 (**G**,**H**)). Lowercase letters indicate significant differences within groups (*p* < 0.05).

Common processing methods such as heating and exposure to light can easily lead to a decrease in anthocyanins content. Therefore, the role of SPI in maintaining AN retention under heated and illuminated conditions was investigated. After the addition of 7S/11S under different processing conditions, the AN retention rates are presented in Figure 1. Figure 1A–D presents that the retention rate of ANs declined progressively as the heating time increased, with pH exerting a pronounced influence on their thermal stability. ANs degraded slowly at pH 3, probably because they existed as stable flavylium cations. At low protein concentrations, the improvement was limited. When the concentrations of 7S and 11S reached 1 mg/mL, the effect was the most pronounced. The retention of ANs increased from 51.6% to 62.3% and 64.7%, respectively. This result aligns with the findings of Chen et al. [29], who reported that the stability of mulberry ANs was enhanced by the addition of whey protein. WPI reduced the thermal degradation rate of ANs by 27.1%. In contrast, at pH 7, free ANs underwent rapid degradation, retaining only 6.2% of initial content after 4 h of heating (Figure 1D). The addition of 7S and 11S significantly slowed down this process. This phenomenon aligned with the results of Wang et al. [30], who found that whey protein improved the thermal stability of C3G at pH 7. Moreover, the protective effect exhibited concentration dependence, as higher protein concentrations progressively reduced the thermal degradation rate of ANs. Similar phenomena could also be observed in other protein–anthocyanin systems, for example, the positive impact of whey protein on mulberry ANs is positively correlated with protein concentration [29].

Figure 1E–H shows the results of light treatment. Compared with heat treatment, the decrease in AN retention was relatively small, indicating that the impact of light on ANs was milder. However, the effects of pH and protein addition on AN stability were consistent with those observed under heating. At pH 3, anthocyanins were structurally stable, and protein addition only slightly improved retention. At pH 7, the degradation rate of free ANs increased significantly, while adding 7S or 11S significantly improved their photostability. This protective effect also increased with protein concentration. A previous study also showed that WPI could significantly slow down the degradation of anthocyanins under light [13].

These results demonstrated that both SPI 7S and 11S enhanced anthocyanin stability under various processing conditions. Further analysis of the stability results revealed differences in the protective effects of 7S and 11S on anthocyanins (Tables S1–S4). Under certain conditions, such as heating at pH 7, 7S was more effective, whereas under conditions of light exposure at pH 3 or 7 and heating at pH 3, 11S was more effective. This could be attributed to conformational differences between 7S and 11S. The structure of 7S is relatively loose, while 11S has a more compact structure and a different distribution of hydrophobic regions. Therefore, the exposed binding sites of the proteins may differ under different pH conditions. Previous studies indicated that protein conformation and hydrophobic surface distribution significantly influenced the binding strength and binding mode with anthocyanin.

### 3.2. Changes in ANs Stability During Simulated Gastrointestinal Digestion

**Figure 2:**
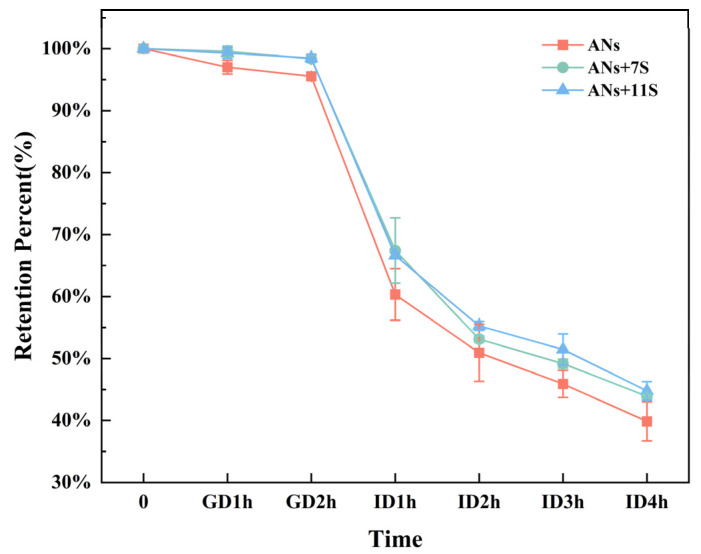
Stability changes in ANs with and without added SPI 7S/11S during digestion.

To investigate changes in AN levels in the stomach and intestines, a simulated digestion process was conducted in vitro, and changes in stability were inferred based on the results. Analysis of the processing stability results showed that stability improved significantly when the anthocyanin-to-protein ratio was 2:1. Furthermore, at pH 7, this ratio yielded the best results after 4 h of heating and 4 days of light exposure; therefore, this ratio was selected for the simulated digestion experiment. As shown in Figure 2, free anthocyanins remained stable during gastric digestion, with a retention rate of 95.5%, while their retention decreased sharply after intestinal digestion (39.8%). This may be attributed to the alkaline intestinal environment that aggravated the instability of ANs. After gastrointestinal digestion, a significant decrease in content can be observed in the AN sample without added protein, which was consistent with the results reported by [31]. Under alkaline conditions, ANs were converted into quinonoidal (A), hemiketal (B) and chalcone (C) forms, thereby limiting their bioavailability. In contrast, after binding with 7S/11S, the retention rates increased to 43.9% and 44.7%, respectively, indicating that 7S/11S improved the stability of ANs in the digestive environment.

### 3.3. Analysis of Fluorescence Quenching Mechanism

**Figure 3:**
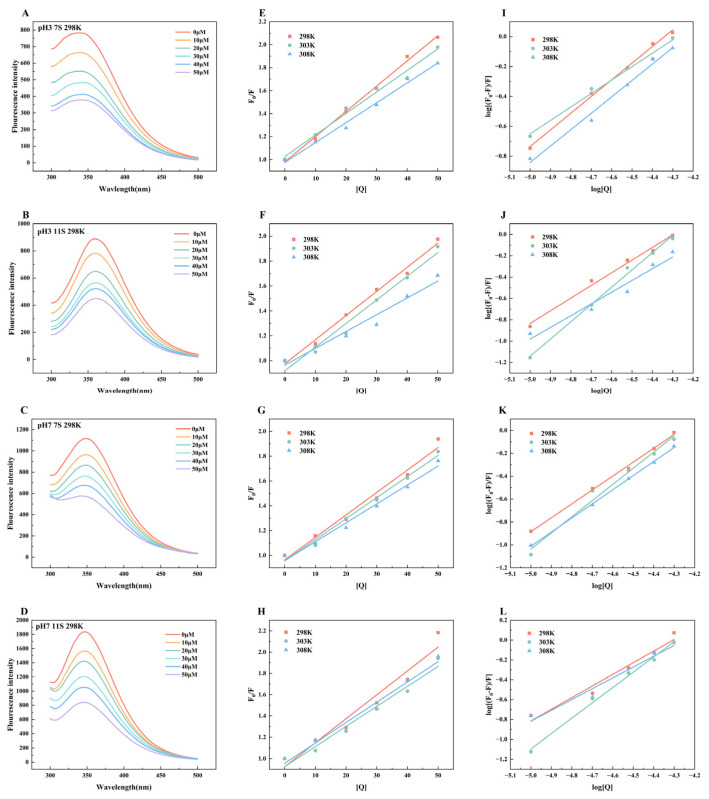
Different pH levels fluorescence emission spectra of SPI 7S/11S in the presence of 0, 10, 20, 30, 40, and 50 μM C3G when excited at 298 K (**A**–**D**); Stern–Volmer plots of SPI 7S/11S with added C3G at 298, 303 and 308 K (**E**–**H**); Double logarithmic regression plots of SPI 7S/11S with added C3G at 298, 303 and 308 K (**I**–**L**).

Fluorescence spectroscopy is commonly used to characterize protein-small molecule interactions [32]. Figure 3A–D shows that under pH 3 and pH 7 conditions, the fluorescence intensities of both 7S and 11S gradually decreased as the C3G concentration increased. This is similar to the findings of Tan et al. [22] regarding the effect of C3G on PPI fluorescence intensity. The fluorescence of 7S and 11S mainly originates from tryptophan residues. These aromatic amino acids are extremely sensitive to the polarity, hydrogen bond network and electrostatic environment of the surrounding microenvironment, and the direction of the emission peak shift directly reflects the changes in their local microenvironment. A slight shift in the emission peak positions of the 7S/11S-C3G complex occurred upon the addition of C3G. At pH 3, the λmax values of 7S and 11S red-shifted to 341 nm and 361 nm, respectively. These results indicate an increase in polarity around the amino acid residues. While the λmax values of 7S and 11S blue-shifted to 342 nm and 345 nm, respectively, at pH 7. These blue shifts suggested that the aromatic residues were located in a more hydrophobic environment. Xin et al. [7] also reported that with increasing C3G concentration, the λmax of β-casein shifted to a longer wavelength at pH 2 and 4, while it shifted to a shorter wavelength at pH 6. These results confirmed that C3G bound tightly to 7S and 11S, forming ground-state complexes and altering the protein microenvironment [33].

A further analysis of the Stern–Volmer model was conducted. Generally, elevated temperatures result in lower complex stability and Stern–Volmer quenching constants (K_sv_), which is characteristic of static quenching. Conversely, the quenching is identified as dynamic quenching [34]. Figure 3E–H presents the curves obtained at 298, 303 and 308 K. As shown in Table 1, the K_SV_ values showed a decrease when the temperature rose under all conditions. The quenching rate constants (K_sv_) of 7S-C3G at 298, 303 and 308 K (pH 3) were approximately 2.194 × 10^12^, 1.817 × 10^12^ and 1.729 × 10^12^ L mol^−1^ s^−1^, respectively. Furthermore, the K_q_ values were much larger than the maximum diffusion-controlled collision rate constant (2.0 × 10^10^ L mol^−1^ s^−1^). These results suggested that static quenching was dominant, resulting from specific protein-C3G interactions rather than collisional encounters [35]. Furthermore, the Ka values of the 7S/11S-C3G complex rose first and then fell with rising temperatures. This phenomenon might be ascribed to a moderate rise in temperature, which promoted favorable intermolecular interactions, while excessively high temperatures disrupted the conformation of protein molecules, resulting in a decrease in the binding affinity between C3G and the complex. The n values approached 1, suggesting that there is a single binding site between proteins and C3G. These results verified the stable binding between C3G and 7S/11S, which was consistent with the viewpoints reported by Zang et al., Lang et al. [12,36].

### 3.4. Thermodynamic Parameters and Types of Binding Forces

Interactions between biomacromolecules and small molecules primarily involve noncovalent forces such as hydrogen bonds. Thermodynamic constants (ΔG, ΔH, and ΔS) can be applied to identify the interaction patterns of binding forces [37]. Previous studies have shown that when both ΔH and ΔS are negative, hydrogen bonding and van der Waals forces are the primary interactions; when both ΔH and ΔS are positive, hydrophobic interactions play the dominant role; and when ΔH < 0 and ΔS > 0, electrostatic forces are predominant [38,39]. As shown in Table 1, the thermodynamic characteristics of the systems differed between pH conditions. At pH 7, the 7S–C3G system showed a ΔH of −12.71 kJ·mol^−1^ and a ΔS of 62.77 J·mol^−1^·K^−1^, suggesting that the primary driving force was electrostatic interaction. In contrast, both ΔH and ΔS values of the 11S–C3G system were negative (−69.73 kJ·mol^−1^ and −126.11 J·mol^−1^·K^−1^), indicating that van der Waals forces and hydrogen bonding dominated the binding process. Under acidic conditions (pH 3), the ΔS and ΔH values of both 7S–C3G and 11S–C3G systems were negative, demonstrating that the complex formation is primarily enthalpy-driven, with hydrogen bonding significantly enhanced and weakened hydrophobic contributions. This can be explained by the fact that C3G exists primarily as a stable flavylium cation at low pH, which increases its molecular polarity and promotes polar interactions. The negative ΔG values for all systems verified that the formation of the complex was spontaneous [40]. 7S is a trimeric protein with a relatively flexible and less compact conformation, which facilitates the exposure of hydrophobic regions, whereas 11S (glycinin) is a hexamer composed of two trimers with a more compact and stable structure and an extensive internal hydrogen bonding network. These structural differences have been well-documented in previous studies [41,42,43,44], which also explains the differences in their thermodynamic parameters. The pH-dependent shift in the type of interaction forces was closely related to the structural transformation of anthocyanins and the adjustment of surface charge distribution of proteins.

### 3.5. CD Spectra of Protein and Protein-C3G Systems

**Figure 4:**
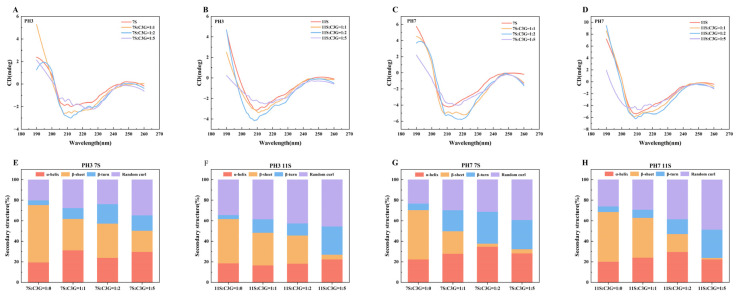
CD spectra of 7S/11S and 7S/11S-C3G complex systems in citric acid buffers of pH 3, 7 (**A**–**D**), and secondary structure contents (**E**–**H**).

Circular dichroism (CD) spectroscopy is a reliable technique for determining the changes in secondary structure that occur in proteins upon binding to small-molecule ligands [45]. Figure 4 showed CD spectra (A–D) and secondary structure content (E–H) of 7S and 11S with different C3G concentrations at different pH levels. At pH 3, adding C3G changed the secondary structure of both proteins. For 7S, the proportions of α-helices, β-turns, and disordered coils increased from 19.5%, 4.5%, and 20.1% to 29.7%, 14.9%, and 34.7%, respectively. The proportion of β-sheets decreased (from 55.8% to 20.7%). The same changes in secondary structure also occur in 11S, and the magnitude of these changes is even more pronounced. These results showed that the binding of C3G disrupted the rigid β-sheet structures within the proteins and promoted the formation of more flexible secondary structural conformations. The increased α-helix content suggested that the overall protein backbone remained ordered, whereas the proteins exhibited greater conformational flexibility, as evidenced by the higher proportions of β-turn and random coil. C3G-mediated binding induced a significant rearrangement of the 7S/11S secondary structure, enhancing structural flexibility while preserving the overall folding characteristics of both proteins. Similar structural alterations have been reported in soybean protein-flavonoid systems, which are regarded as typical characteristics of ligand binding-induced protein conformational adjustment [26]. At pH 7, the trend of secondary structure changes in SPI 7S/11S was consistent with that at pH 3, namely, with the exception of the proportion of β-sheets, which has decreased, the proportions of all other structures have increased. Therefore, the conformational adjustments induced by C3G binding provide a structural basis for the formation of complexes and enhance their stability.

### 3.6. FT-IR Spectroscopy Analysis

**Figure 5:**
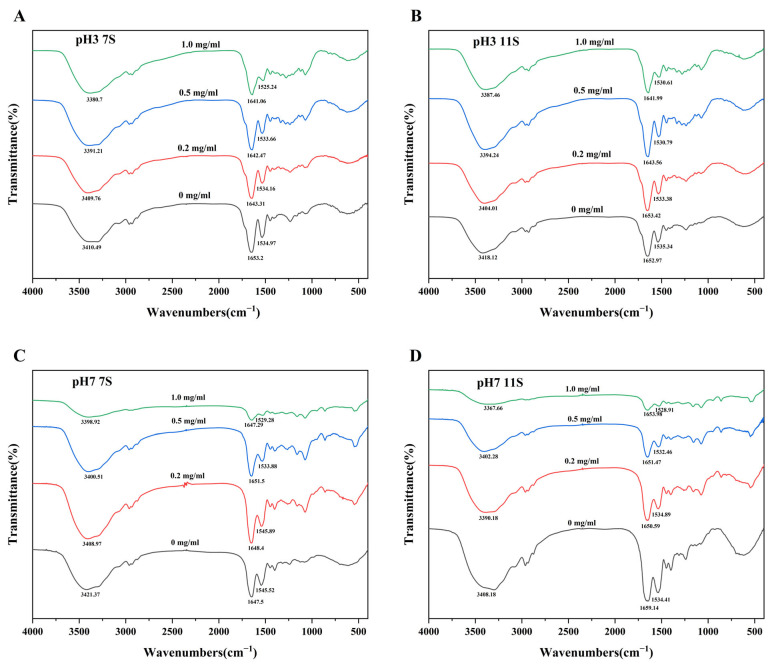
FT-IR spectra of 7S and 7S-C3G complex systems at pH 3 (**A**) and pH 7 (**C**). FT-IR spectra of 11S and 11S-C3G complex systems at pH 3 (**B**) and pH 7 (**D**).

FT-IR spectroscopy is extensively acknowledged as a powerful tool for monitoring and assessing conformational and secondary alterations in proteins [46]. Figure 5 showed the FT-IR spectra of 7S and 11S upon C3G addition at various concentrations under different pH conditions. The formation of complexes resulted in modifications to their transmittance and peak positions. At different pH values, C3G addition induced varying degrees of blue shifts and red shifts in the Amide bands of 7S and 11S.

At different pH levels, the characteristic peaks of 7S/11S in the Amide A band gradually red-shifted as C3G concentration increased. The peaks shifted from 3410/3418 cm^−1^ and 3421/3408 cm^−1^ to 3380/3387 cm^−1^ and 3398/3367 cm^−1^, respectively, with an obvious broadening of the peak shape. This confirmed the existence of intermolecular hydrogen bonds in 7S/11S-C3G complexes, which was associated with the O–H stretching vibrations of the complexes [47]. Meanwhile, changes in peak intensity and slight red shifts were detected in the Amide II band, further verifying the rearrangement of the hydrogen bond network within the protein main chain. While the CD results provided a quantitative breakdown of secondary structure alterations, the FT-IR spectra further verified these transitions at the functional group level. In conjunction with the CD results, it was confirmed that the binding of C3G induces a more flexible protein conformation through the rearrangement of the internal hydrogen-bond network.

These spectral shifts also indicated conformational alterations in the secondary structure of proteins [46]. The secondary structures of 7S and 11S in the sample were quantified using Gaussian deconvolutions performed with PeakFit v4.12. Specific changes in the content of secondary structures are shown in Tables S5 and S6.

### 3.7. Analysis of Molecular Docking

**Figure 6:**
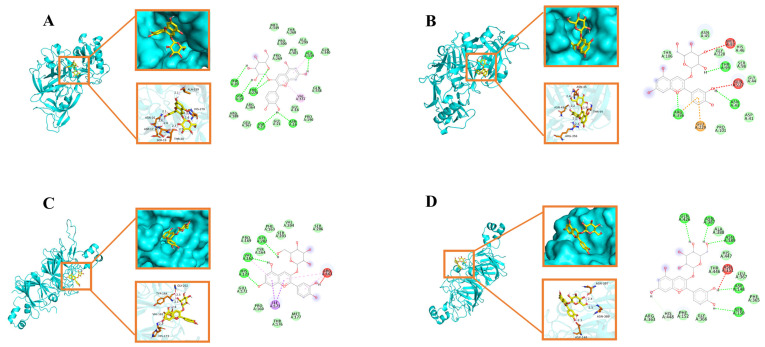
The 3D structure, the Solvent Accessible Surface (SAS) and detailed binding mode of 7S-C3G at pH 3 (**A**) and pH 7 (**B**), The 3D structure, electrostatic surface and detailed binding mode of 11S-C3G at pH 3 (**C**) and pH 7 (**D**) (The large cyan areas in the image represent the SAS of the proteins).

Molecular docking is a computer-based simulation technique employed to investigate interactions between proteins and small molecule ligands [48]. Molecular docking was performed to explore the binding mechanism between C3G and 7S/11S under different pH conditions (Figure 6A–D).

At pH 3, C3G was able to access the binding pocket of SPI 7S (Figure 6A). C3G established hydrogen bonds with multiple amino acid residues, including Asn, Thr, Arg, and Ser. In addition, van der Waals interactions and π-alkyl contacts were observed. These interactions allowed C3G to be embedded in the hydrophobic cavity of the protein, which improved the binding stability. Previous studies have shown that hydrogen bonding and van der Waals forces are the main drivers of the interaction between C3G and 7S. Residues such as Asn69 and Thr101 were identified as key binding sites, and they contributed significantly to the binding energy [49]. C3G could also enter the binding pocket of 11S. C3G formed hydrogen bonds and van der Waals interactions with residues such as Thr, Pro, Glu, and Ser. In addition, π-sigma and π-alkyl interactions were observed. Previous studies reported that Thr82 and Pro86 are important residues involved in the interaction between 11S and C3G [49].

At pH 7, C3G still interacted with both 7S and 11S proteins (Figure 6C,D), but the interaction pattern changed. In the C3G–7S complex, interactions between π-cations and π-anions, in addition to hydrogen bonds and van der Waals forces, had also been observed. C3G could still diffuse into the binding cavity of 11S and interact with residues such as Asn, Thr, Ser, and His through hydrogen bonds and van der Waals forces. The binding energies of the complexes are shown in Table S7. At pH 3.0, the 11S–C3G system had the lowest binding energy (−7.9 kcal/mol). This value was better than that of the 7S–C3G system (−7.4 kcal/mol). This confirmed at the molecular level that 11S had a stronger protective effect on anthocyanins, as observed in experiments. At pH 7.0, the 7S–C3G system showed a binding energy of −7.6 kcal/mol, and the 11S–C3G system showed −7.4 kcal/mol. Each system also exhibited stable binding affinity. This supported the spontaneous formation of the complexes.

To provide direct calorimetric evidence for the binding process, ITC experiments were conducted (Figure S5 and Table S8). The negative ΔG values obtained from ITC (−25.50 and −28.65 kJ/mol) confirmed the spontaneous nature of the binding, mirroring the trends observed in fluorescence quenching. The K_d_ value for 11S (9.52 × 10^−6^ M) was significantly lower than that for 7S (3.40 × 10^−5^ M), corroborating the molecular docking results that 11S possesses a more favorable binding affinity for C3G. Furthermore, the thermodynamic profile showed that both ΔH < 0 and ΔS > 0 for the systems. This combination identifies electrostatic interactions as the predominant driving force, which is further complemented by hydrogen bonding and the desolvation of the binding pocket (contributing to the positive ΔS) [38,39]. The stoichiometry (n) values of 1.21–1.36 suggest that while a primary specific pocket is occupied, the flexibility induced by binding may also facilitate secondary non-specific surface associations.

These findings indicated that C3G could enter the binding pockets of both 7S and 11S at pH 3 and pH 7, forming stable complexes. The binding was mainly driven by hydrogen bonds, hydrophobic interactions, and van der Waals forces, along with other weak interactions. The binding between 11S and C3G was stronger than that between 7S and C3G. Some studies have reported that 11S usually shows stronger binding ability with polyphenols than 7S. Zhang et al. [50] found that the affinity of piperine for 11S protein was higher than that for 7S protein. Therefore, these results suggest that, in most cases, 11S may provide better stabilization for anthocyanins, which aligns with the processing stability results.

## 4. Conclusions

In this research, the processing stability of ANs was enhanced through complex formation with SPI 7S and 11S. To elucidate the interaction mechanisms of the system, multispectral approaches combined with molecular docking were applied. The results showed that both 7S and 11S increased the retention of ANs under light and heat treatments at pH 3 and pH 7 to different extents. Under heating at pH 3.0 (4 h), the retention of ANs increased significantly from 51.6% to 62.3% (7S) and 64.7% (11S). This effect was also confirmed during simulated digestion, where 11S addition maintained a higher retention of 44.7% compared to the control (39.8%). Fluorescence and thermodynamic analysis revealed that 11S possessed a stronger binding affinity for C3G (K_a_= 1.2770 × 10^5^ L/mol, ΔG = −33.15 kJ/mol at pH 3.0) than 7S. Fluorescence spectroscopy showed that the presence of C3G results in a decrease in protein fluorescence intensity and the occurrence of static quenching. At the same time, the polarity of the microenvironment surrounding the amino acid residues changed. Thermodynamic analysis suggested that hydrogen bonding and hydrophobic interactions were the primary driving forces behind the reaction. Circular dichroism and FT-IR results revealed that C3G binding induced modifications in the secondary structure of the proteins. The protein structure became more flexible after binding. Molecular docking further showed that C3G could bind with 7S and 11S through multiple non-covalent interactions to form stable complexes. This study demonstrated that SPI 7S and 11S can enhance the stability of *Lonicera caerulea* L. anthocyanins. and preliminarily revealed its potential mechanism of action. These findings offer valuable insights into enhancing anthocyanin stability and facilitating their effective application in food systems.

Despite the systematic insights provided by this study, several limitations warrant further investigation. First, the current research was conducted in a controlled buffer system; however, the presence of complex ingredients in real food products might interfere with the 7S/11S–ANs interactions. At the same time, the impact of time can also be taken into account. Second, future research could also utilize molecular dynamics (MD) simulations to further investigate dynamic stability. At the same time, future discussions could explore the sustainability of using plant-based proteins such as SPI as eco-friendly carriers and conduct a more comprehensive assessment of their environmental and economic performance.

## Figures and Tables

**Table 1:** Quenching constants (K_sv_, K_q_ and K_a_), number of binding sites (n), and thermodynamic parameters (ΔH, ΔS and ΔG) at various temperatures (298 K, 303 K, and 308 K) and pH (3, 7).

	T (K)	K_sv_ (×10^3^ L·mol^−1^)	K_q_ (×10^11^ L·mol^−1^s^−1^)	K_a_ (×10^5^ L·mol^−1^)	n	ΔH (kJ⋅mol^−1^)	ΔS (J⋅mol^−1^K^−1^)	ΔG (kJ⋅mol^−1^)
pH3 7S-C3G	298	21.94	21.94	0.7075	1.1171	−37.553	−38.792	−25.993
	303	18.71	18.71	0.0709	0.9004			−25.798
	308	17.29	17.29	0.4424	1.0977			−25.605
pH3 11S-C3G	298	19.37	19.37	1.2770	1.1881	−99.219	−221.701	−33.152
	303	18.96	18.96	90.564	1.6198			−32.044
	308	13.49	13.49	0.3295	1.0999			−30.935
pH7 7S-C3G	298	18.12	18.12	1.7120	1.2209	−12.712	62.768	−31.417
	303	17.02	17.02	10.6358	1.4127			−31.731
	308	15.26	15.26	1.4190	1.2326			−32.045
pH7 11S-C3G	298	22.46	22.46	1.1365	1.1741	−69.732	−126.107	−32.153
	303	18.82	18.82	41.576	1.5419			−31.522
	308	19.08	19.08	0.4358	1.0914			−30.892

## Data Availability

The data presented in this study are available on request from the corresponding author. The data are not publicly available due to privacy concerns.

## Supplementary Materials

The following supporting information can be downloaded at https://www.mdpi.com/article/10.3390/foods15111933/s1, Figure S1: SDS-PAGE of 7S and 11S; Figure S2: UV absorption spectra of C3G at different pH values; Figure S3: The fluorescence spectra of SPI7S (A, B) and 11S (C, D) at different concentrations of C3G in pH3 at 303 K and 308 K; Figure S4: The fluorescence spectra of SPI7S (A, B) and 11S (C, D) at different concentrations of C3G in pH7 at 303 K and 308 K; Figure S5: Combined heat flux–time curves for C3G-7S (A) and C3G-11S (B). Enthalpy–concentration curves and isotherms for C3G-7S (C) and C3G-11S (D); Table S1: At pH 7, retention rate of anthocyanins after heating; Table S2: At pH 3, retention rate of anthocyanins after heating; Table S3: At pH 7, anthocyanins retention rate after light exposure; Table S4: At pH 3, anthocyanins retention rate after light exposure; Table S5: At pH 3, the content of the secondary structure on FT-IR; Table S6: At pH 7, the content of the secondary structure on FT-IR; Table S7: Binding energies of 7S and 11S to C3G at pH 3 and pH 7; Table S8: ITC-derived thermodynamic parameters.
