# Supplementary material for: The Influence of SPI 7S and 11S on the Stability of Lonicera caerulea L. Anthocyanins and Interaction Mechanism with C3G
Source: Foods. 2026 May 29;15(11):1933. doi: 10.3390/foods15111933 (PMC13256205; doi:10.3390/foods15111933)
Supplement: Supplementary file 1 [file foods-15-01933-s001.zip › foods-4274738-supplementary.pdf]

foods

MDPI

Supplementary material

The Influence of SPI 7S and 11S on the Stability of *Lonicera caerulea* L. Anthocyanins and Interaction Mechanism with C3G

Yingying Zhou [1], Yixin Yuan [1], Zhicong Wang [1], Di Wu [1,2], Yinan Du [1,2,*] and Jiangning Hu [1,2,*]

[1] State Key Laboratory of Marine Food Processing and Safety Control, Dalian Polytechnic University, Dalian 116034, China; 15238225546@163.com (Y.Z.); yxyuan20@mails.jlu.edu.cn (Y.Y.); 18237138753@163.com (Z.W.); m13039998695@163.com (D.W.)
[2] National Engineering Research Center of Seafood, School of Food Science and Technology, Dalian Polytechnic University, Dalian 116034, China
* Correspondence: dyn7381@163.com (Y.D.); hujiangning2005@hotmail.com (J.H.); Tel.: +86-411-86332275

Figure S1 SDS-PAGE of 7S and 11S.

Figure S2 UV absorption spectra of C3G at different pH values.

Figure S3 The fluorescence spectra of SPI7S (A, B) and 11S (C, D) at different concentrations of C3G in pH3 at 303K and 308.

Figure S4 The fluorescence spectra of SPI7S (A, B) and 11S (C, D) at different concentrations of C3G in pH7 at 303K and 308K.

Figure S5 Combined heat flux-time curves for C3G-7S (A) and C3G-11S (B). Enthalpy-concentration curves and isotherms for C3G-7S (C) and C3G-11S (D).

Table S1 At pH 7, retention rate of anthocyanins after heating.

Table S2 At pH 3, retention rate of anthocyanins after heating.

Table S3 At pH 7, anthocyanins retention rate after light exposure.

Table S4 At pH 3, anthocyanins retention rate after light exposure.

Table S5 At pH3, the content of the secondary structure on FT-IR.

Table S6 At pH7, the content of the secondary structure on FT-IR.

Table S7 Binding energies of 7S and 11S to C3G at pH 3 and pH 7.

Table S8 ITC-derived thermodynamic parameters.

 https://doi.org/10.3390/foods15111933

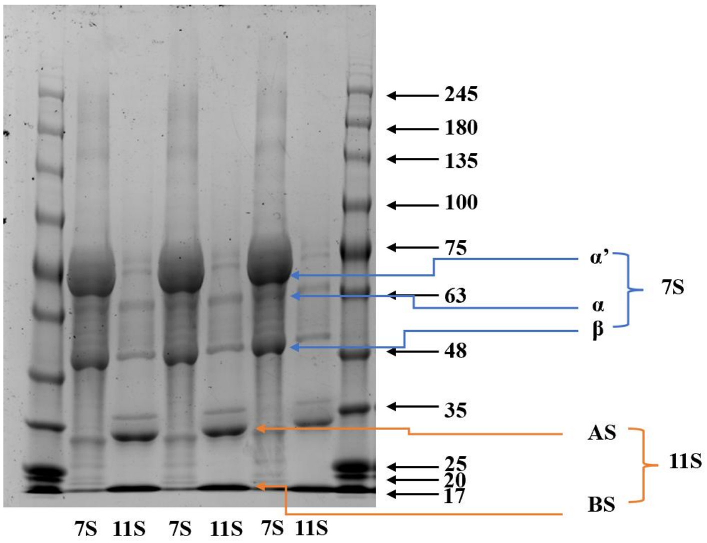

Figure S1: SDS-PAGE of 7S and 11S

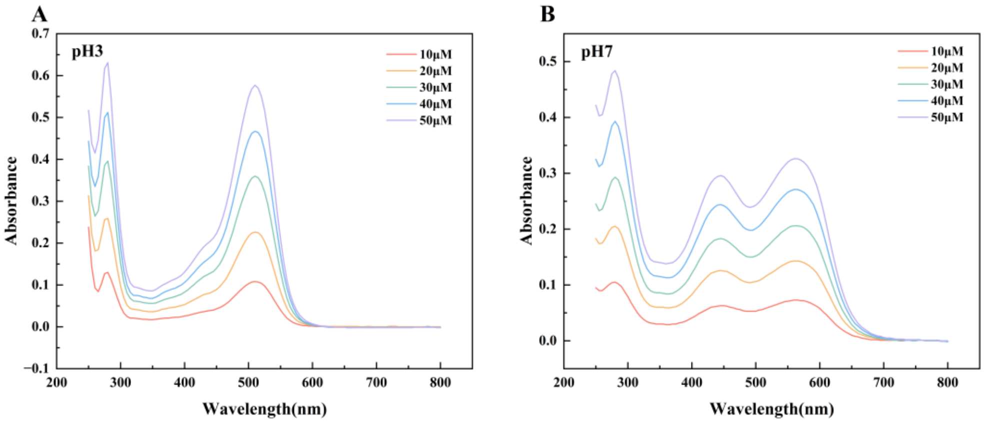

Figure S2: UV absorption spectra of C3G at different pH values.

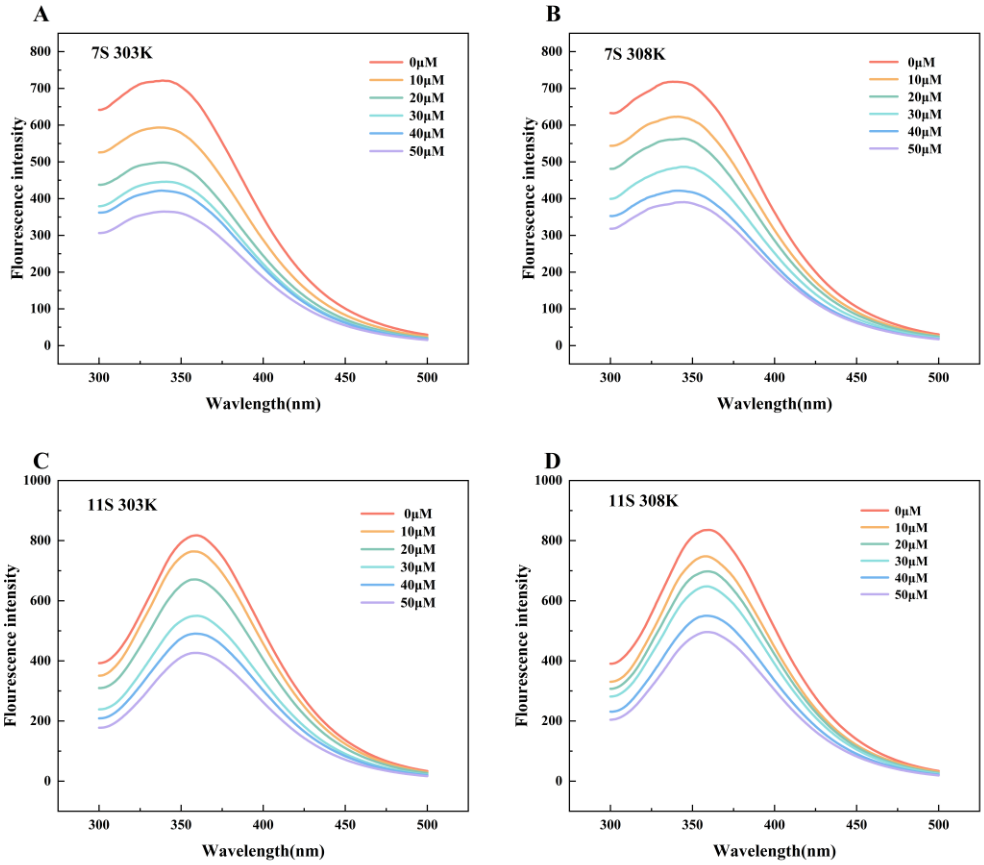

Figure S3: The fluorescence spectra of SPI7S (A, B) and 11S (C, D) at different concentrations of C3G in pH3 at 303K and 308.

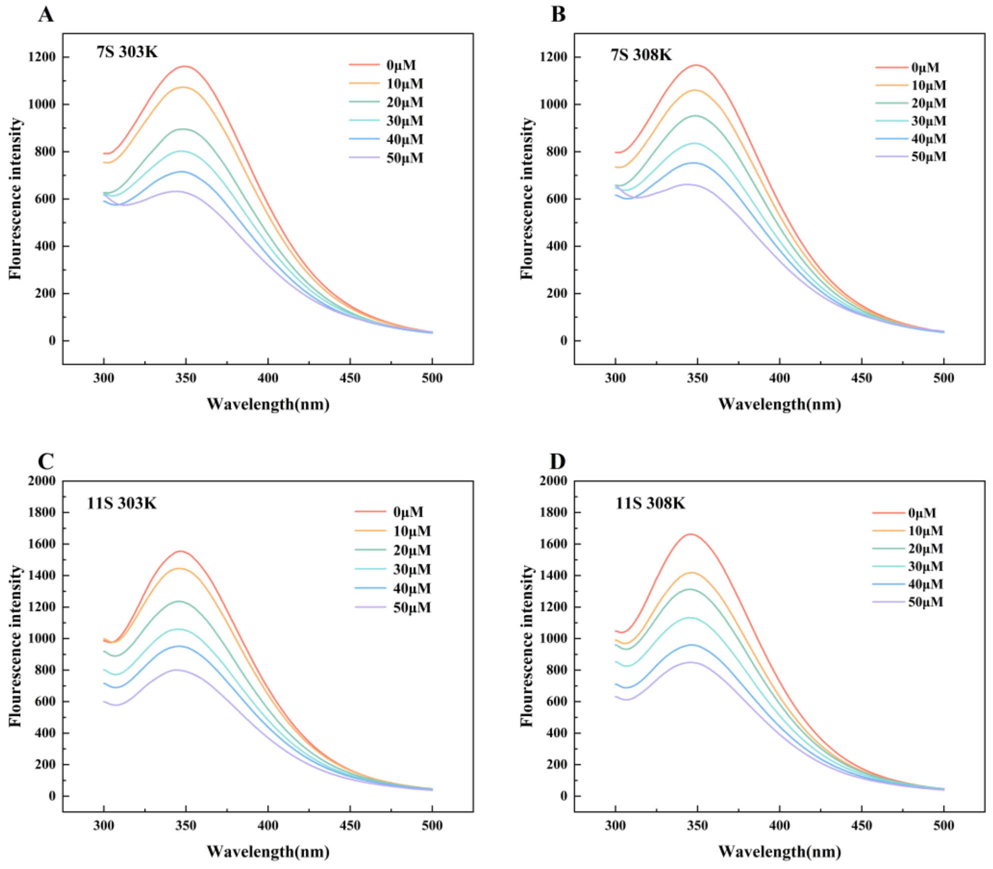

Figure S4: The fluorescence spectra of SPI7S (A, B) and 11S (C, D) at different concentrations of C3G in pH7 at 303K and 308K.

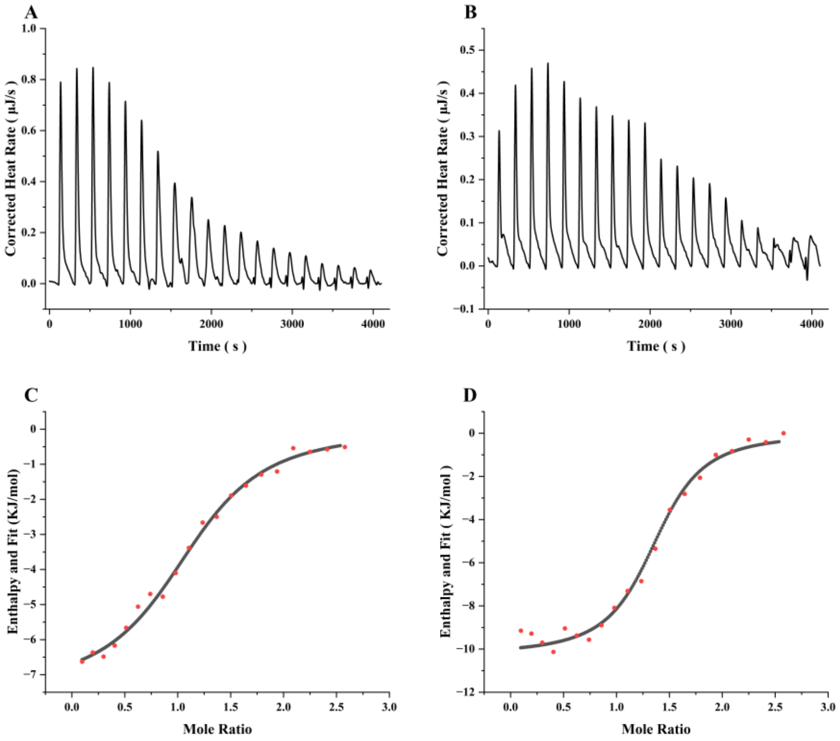

Figure S5: Combined heat flux-time curves for C3G-7S (A) and C3G-11S (B). Enthalpy-concentration curves and isotherms for C3G-7S (C) and C3G-11S (D)

Table S1: At pH 7, retention rate of anthocyanins after heating

Time (h)	sample	0.1mg/ml	0.2mg/ml	0.5mg/ml	1.0mg/ml
1	7S	32.8±0.8%	35.6±0.7%	41.7±1.7%	38.4±1.4%
	11S	30.9±0.5%	32.7±1.4%	38.7±1.4%	33.8±1.8%
2	7S	19.5±1.8%	22.7±1.6%	25.2±1.1%	25.1±1.7%
	11S	20.1±0.4%	21.7±0.4%	24.6±2.1%	22.1±1.6%
3	7S	12.9±0.7%	13.2±1.6%	14.7±0.5%	16.7±0.9%
	11S	10.7±1.4%	14.0±0.8%	15.4±1.4%	15.4±0.8%
4	7S	7.1±0.6%	7.2±0.3%	7.5±0.5%	7.4±0.6%
	11S	6.7±0.2%	7.4±0.5%	8.1±0.7%	7.2±0.6%

Table S2: At pH 3, retention rate of anthocyanins after heating

Time (h)	sample	0.1mg/ml	0.2mg/ml	0.5mg/ml	1.0mg/ml
1	7S	92.8±2.9%	91.9±3.3%	90.4±0.9%	91.3±1.3%
	11S	90.3±1.3%	93.1±3.4%	94.5±2.4%	95.1±2.3%
2	7S	77.9±3.1%	81.6±0.9%	77.7±2.3%	85.5±4.5%
	11S	78.7±2.9%	80.4±2.7%	79.9±2.8%	85.9±0.5%
3	7S	64.2±3.5%	72.2±3.2%	68.9±0.4%	73.8±4.5%
	11S	67.2±3.6%	67.9±1.8%	71.9±3.1%	73.3±3.5%
4	7S	51.1±1.1%	59.1±2.8%	61.3±1.3%	62.3±3.2%
	11S	53.8±3.6%	60.1±2.7%	61.0±3.1%	64.7±3.7%

Table S3: At pH 7, anthocyanins retention rate after light exposure

Time (d)	sample	0.1mg/ml	0.2mg/ml	0.5mg/ml	1.0mg/ml
1	7S	88.0±2.3%	88.5±0.8%	91.5±1.7%	90.8±1.8%
	11S	89.3±0.9%	92.1±0.9%	89.8±1.4%	90.8±1.7%
2	7S	82.4±0.9%	83.8±2.4%	85.2±0.6%	85.6±0.8
	11S	79.8±1.5%	82.2±1.4%	84.5±1.8%	87.2±0.6%
3	7S	74.2±1.5%	69.6±0.5%	69.3±0.6%	74.3±1.2%
	11S	72.4±2.6%	74.1±1.4%	71.2±1.8%	74.6±2.1%
4	7S	53.6±1.8%	55.6±2.1%	61.3±2.3%	59.9±1.9%
	11S	62.3±2.5%	62.5±2.1%	56.3±1.9%	60.5±0.3%

Table S4: At pH 3, anthocyanins retention rate after light exposure

Time (d)	sample	0.1mg/ml	0.2mg/ml	0.5mg/ml	1.0mg/ml
1	7S	89.5±1.3%	84.1±1.2%	85.1±1.6%	86.0±0.7%
	11S	89.2±1.7%	92.4±4.1%	91.5±3.3%	89.5±3.9%
2	7S	85.9±0.3%	83.8±1.6%	82.9±2.0%	84.5±0.5%
	11S	86.6±2.6%	85.4±0.8%	80.4±1.6%	83.8±0.5%
3	7S	77.4±2.0%	78.3±0.6%	75.6±0.5%	79.3±2.1%
	11S	76.5±3.5%	77.1±2.4%	74.7±3.7%	77.5±1.4%
4	7S	64.4±0.7%	64.7±0.5%	66.7±2.0%	68.9±1.9%
	11S	65.3±1.3%	65.9±0.7%	67.2±1.0%	69.9±0.8%

Table S5: At pH3, the content of the secondary structure on FT-IR.

Samples	α-Helix (%)	β-Sheet (%)	β-Turn (%)	Random Coil (%)
7S	16.61	33.29	33.21	16.89
7S-$C3G_{0.2}$	16.82	32.66	33.45	17.07
7S-$C3G_{0.5}$	17.18	31.65	33.92	17.25
7S-$C3G_{1.0}$	19.29	27.95	35.44	17.32
11S	14.49	27.45	28.58	29.49
11S-$C3G_{0.2}$	22.21	26.44	29.20	22.15
11S-$C3G_{0.5}$	30.43	24.77	29.02	15.78
11S-$C3G_{1.0}$	17.63	31.03	34.05	17.29

Table S6: At pH 7, the content of the secondary structure on FT-IR

Samples	α-Helix (%)	β-Sheet (%)	β-Turn (%)	Random Coil (%)
7S	30.78	23.88	29.64	15.70
7S-C3G$_{0.2}$	32.45	20.84	31.56	15.15
7S-C3G$_{0.5}$	32.18	20.89	31.92	15.01
7S-C3G$_{1.0}$	16.25	21.45	30.42	31.88
11S	15,35	33.42	34.05	17.18
11S-C3G$_{0.2}$	16.54	29.37	36.44	17.65
11S-C3G$_{0.5}$	17.48	28.91	36.16	17.45
11S-C3G$_{1.0}$	16.01	20.42	48.57	15.00

Table S7 Binding energies of 7S and 11S to C3G at pH 3 and pH 7.

Sample	Affinity (kcal/mol)
pH 3 7S-C3G	-7.4
pH 3 11S-C3G	-7.9
pH 7 7S-C3G	-7.6
pH 7 11S-C3G	-7.4

Table S8: ITC-derived thermodynamic parameters.

Sample	Model	K_d (M)	n	ΔH(kJ/mol)	ΔS(J/mol·K)	ΔG(kJ/mol)
C3G-7S	Independent	3.40×10^{-5}	1.205	-7.258	61.22	-25.502
C3G-11S	Independent	9.52×10^{-6}	1.358	-10.19	61.94	-28.648
